# Acute Submandibular Sialadenitis—A Case Report

**DOI:** 10.1155/2012/615375

**Published:** 2012-07-24

**Authors:** Rakhi Chandak, Shirish Degwekar, Manoj Chandak, Shivlal Rawlani

**Affiliations:** ^1^Department of Oral Medicine and Radiology, Sharad Pawar Dental College and Hospital, Datta Meghe Institute of Medical Sciences University, Maharashtra, Sawangi (M), Wardha 442004, India; ^2^Department of Conservative Dentistry, Sharad Pawar Dental College and Hospital, Datta Meghe Institute of Medical Sciences University, Maharashtra, Sawangi (M), Wardha 442004, India

## Abstract

Many conditions affect the salivary glands. Acute sialadenitis is infectious or inflammatory disorders of the salivary glands. The exact frequency of submandibular sialadenitis is unclear. The acute conditions more typically involve the parotid and submandibular glands. During an acute inflammatory process, there is swelling of the affected gland, overlying pain, gland tenderness, fever, and on occasion difficulty in opening the mouth. Initial treatment should include rehydration oral antistaphylococcal antibiotic should be started while awaiting culture results. Hygiene and repeated massaging of the gland when tenderness had subsided. The present report describes a case of acute submandibular sialadenitis in a 70-year-old female.

## 1. Introduction

Many conditions affect the salivary glands. They affect all of the salivary tissues, but all conditions affect the parotid and the submandibular glands preferentially because of their size and location. Adults and children are commonly affected [1]. Sialadenitis of the submandibular gland is a relatively commonly encountered yet infrequently discussed topic. Causes range from simple infection to autoimmune etiologies, although not as frequent as sialadenitis of the parotid gland [2]. 

Acute sialadenitis is infectious or inflammatory disorders of the salivary glands [3]. The exact frequency of submandibular sialadenitis is unclear. The incidence of acute suppurative parotitis has been reported at 0.01–0.02% of all hospital admissions. The submandibular gland is suggested to account for approximately 10% of all cases of sialadenitis of the major salivary glands. No race, age and sex predilection per se exists. sialadenitis as a whole tends to occur in the older, debilitated, or dehydrated patient [2]. 

The acute conditions more typically involve the parotid and submandibular glands. During an acute inflammatory process, there is swelling of the affected gland, overlying pain, gland tenderness, fever, and on occasion difficulty in opening the mouth. Often the pain is intensified with eating in that food ingestion stimulates saliva flow, which will typically cause the gland to swell and thus exacerbate the preexisting symptoms. Acute inflammatory processes largely fall into bacterial, viral, and autoimmune states. In chronic gland disorder, the symptoms are similar, although much less intense. In the inflammatory conditions, the gland is not so much a target of bacterial or viral processes but is inflamed by antibodies directed against salivary gland tissues [3].

Initial treatment should include rehydration, improved oral antistaphylococcal antibiotic should be started while awaiting culture results. Hygiene and repeated massaging of the gland when tenderness had subsided [1]. The present report describes a case of acute submandibular sialadenitis in a 70-year-old female.

## 2. Case Report

A 70-year-old female patient was referred to department of Oral Medicine and Radiology with a chief complaint of a swelling in left side of neck since 12 days and pain in swelling since 10 days. Pain was increased in intensity while swallowing. Patient gives no history of fever and difficulty in eating and speaking. Patient noticed that initially swelling was initially small in size and gradually increase to present size of 4-3 cm. The patient's medical history was unremarkable.

Clinical examination revealed that spherical shape swelling was present and that measured 4-3 cm in diameter. Swelling extending from 1 cm below lower border of mandible to upper border of thyroid cartilage. Swelling has well-defined and regular border, surface was smooth and skin over the swelling was red and shiny. It was tender on palpation but temperature was not raised. Consistency of swelling was soft and rubbery and fluctuation was present but it was not fixed to overlying skin. Other intraoral findings were grossly carious lower left second molar and fracture crown with right and left first molar. Considerable deposition of sub- and supragingival calculus and stains was noticed. Missing teeth were upper right and left molars.

**Figure 1:**
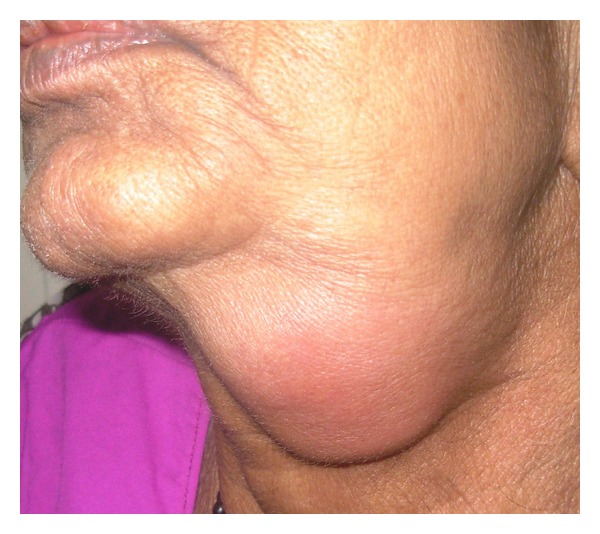
Clinical extraoral photograph of swelling in submandibular region on left side.

When swelling is seen at the side of neck, it is important to formulate the differential diagnosis since this would help further evaluation of the condition and management of the patient. After considering all clinical findings following entities were considered in differential diagnosis—acute submandibular sialadenitis and benign swelling of neck (Figure 1).

**Figure 2:**
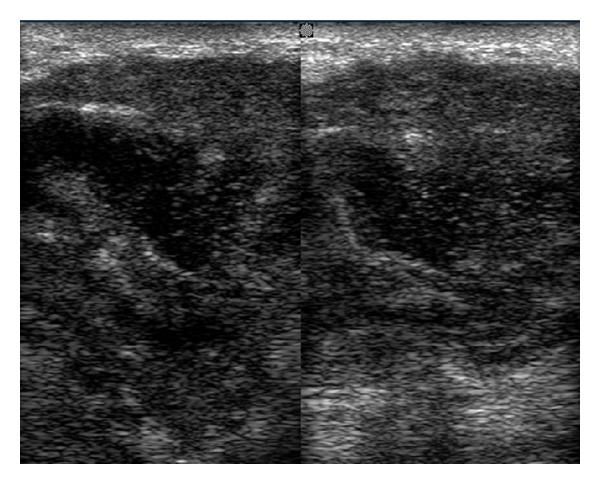
Ultrasonographic findings ill defined hypoecoic lesion.

**Figure 3:**
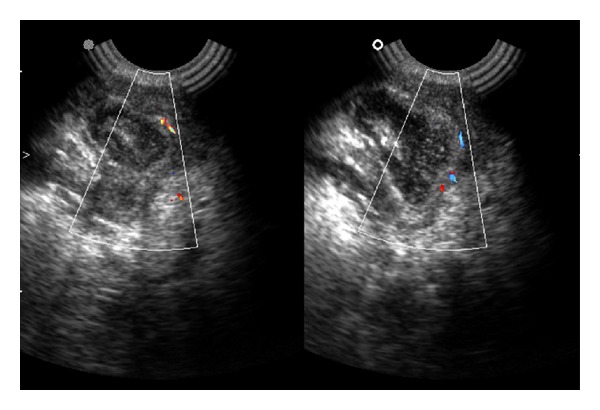
Color Doppler findings shows increased vascularity.

**Figure 4:**
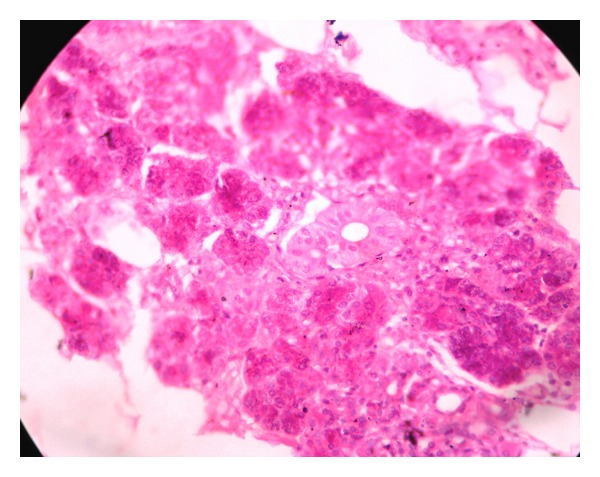
Photomicrograph (40x) of submandibular sialadenitis.

After that patient was advised for drainage of abscess. The investigatory work up included complete hemogram, intra oral radiographs, orthopantomograph and ultrasonography. Routine hematological investigations were within normal limit. Orthopantomograph shows carious root fracture with lower left second molar and advanced mesial caries with periapical radiolucency with lower left third molar. Ultrasonographic findings of swelling were lobular in shape, ill-defined hypoechoic lesion with heterogenous ultrasound architecture of lesion. Posterior echoes were unchanged, ultrasound characteristic of tissues were solid and no any calcification was observed. Ultrasonographic impression was enlarged submandibular gland with focal abscess suggestive of submandibular abscess or sialadenitis (Figures 2 and 3). Incision and drainage was performed. Adequate hydration should be ensured and electrolyte imbalances corrected with the administration of a single dose of parenteral antibiotics, followed by oral antibiotics for a period of 5–7 days. Amoxycillin clavulanic acid (625 mg) is an excellent choice and provides good coverage against typical organisms. Patient was called for follow-up visit of 3 days from the first visit and then 1 week later. (with improvement). After that lower left first and second molar and right first molar were extracted (because patient was not willing for conservative approach) as it can cause recurrence of infection. Specimen sent for histopathological examination. The biopsy report was interpreted as an acute submandibular sialadenitis as H&E section revealed vasodilatation and increasing numbers of neutrophils in the submandibular vessels, emigrating into the parenchyma and filling ducts. Colonies of bacteria may also be seen particularly in the ducts. The ducts become dilated and filled with neutrophils; duct epithelium and then acini are progressively destroyed, leading to formation of microabscesses and destruction of large areas of the gland (Figure 4). Thus, a final diagnosis of acute submandibular sialadenitis was given. There is no residual or recurrent, swelling apparent in the area of biopsy after a follow-up period of 6 months.

## 3. Discussion

A variety of factors affect the susceptibility of the different salivary glands to bacterial infection but among the most important are their rates of salivary flow, the composition of their saliva, and variations in or damage to their duct systems [4]. Raad et al. (1990) have drawn attention to and reviewed reports of this entity of which there were 12 cases among their 29 patients with acute bacterial sialadenitis. Unlike suppurative parotitis, sialolithiasis was an important predisposing factor but xerostomia was also common [4].

Clinically, acute submandibular sialadenitis differs from parotitis mainly in the site of the swelling and discharge of pus from Wharton's duct. A wide variety of bacteria has been incriminated, but *Staphylococcus aureus *has been the most frequently reported isolate [5]. The other isolated organisms have included streptococci, *Pseudomonas aeruginosa, Escherichia coli* and *Moraxella catarrhalis.*

The diagnosis of submandibular sialadenitis can be made on clinical grounds, submandibular sialadenitis takes several forms. The diagnostic workup of any submandibular enlargement begins with a thorough history. However, systemic manifestations may be minimal.

Examination with ultrasound is noninvasive, cheap, and useful for diagnosis, differential diagnosis and excluding the other predisposing factors like anatomical abnormalities of Wharton's duct, mechanical salivary duct obstruction secondary to a sialolith and infection related to a submandibular gland neoplasm; however, in our case, patient had bacterial infection of submandibular salivary gland [5].

 The administration of antimicrobial therapy is an essential part of the management of patients with suppurative sialadenitis. Most cases respond to antimicrobial therapy; however, sometimes abscess formation requires surgical drainage [6].

In the acute viral and the vast majority of acute bacterial infections, the gland returns to an asymptomatic state. Certain individuals with chronic bacterial infections not responding to appropriate conservative and antibiotic measures may require either radiation or removal of the affected gland to control its symptoms.The prognosis of acute sialadenitis is very good. Most cases are easily treated with conservative medical management, and admission is the exception, not the rule. Acute symptoms resolve within 1 week; however, edema in the area may last for several weeks [3].

## 4. Conclusion

Patients with any form of sialadenitis should be educated as to the value of hydration and excellent oral hygiene. This lessens the severity of the attacks and prevents dental complications. Patients with sialadenosis should be educated regarding the mechanism of their underlying pathology and methods of maintaining control over them [7, 8].
